# Evaluation of Oxidative Stress in Patients with Difficult-to-Heal Skin Wounds Treated with Hyperbaric Oxygen

**DOI:** 10.1155/2020/1835352

**Published:** 2020-07-31

**Authors:** Jarosław Paprocki, Marta Pawłowska, Paweł Sutkowy, Jacek Piechocki, Alina Woźniak

**Affiliations:** ^1^Department of Medical Biology and Biochemistry, Collegium Medicum of Nicolaus Copernicus University, Karłowicza 24, 85-092 Bydgoszcz, Poland; ^2^Mazovian Centre for Hyperbaric Therapy and Wound Treatment in Warsaw, Wołoska 137, bud. “O”, 02-507 Warszawa, Poland

## Abstract

**Objective:**

To determine the concentration of thiobarbituric acid reactive substances (TBARS) in erythrocytes and blood plasma, and the activities of selected antioxidant enzymes: catalase (CAT), superoxide dismutase (SOD), and glutathione peroxidase (GPx) in erythrocytes in patients receiving hyperbaric oxygen (HBO) treatment due to difficult-to-heal “skin wounds”. *Material and Methods*. Indices of oxidative stress were assessed in venous blood taken from 23 patients three times: immediately before HBO procedure, approx. 5 minutes after leaving the hyperbaric chamber, and after 25 HBO procedures. Moreover, selected blood counts were measured in the collected material two times: prior to treatment and after 25 HBO procedures.

**Results:**

A statistically significant positive correlation between the CAT activity and the TBARS concentration in the erythrocytes of patients was found before treatment in the hyperbaric chamber (*r* = 0.394; *P* ≤ 0.05). No statistically significant changes in the TBARS concentration in erythrocytes and blood plasma were observed both after the first HBO procedure and after 25 procedures. No statistically significant changes in the activities of CAT, SOD, and GPx were noted. Platelet count decreased by 18.7% (*P* ≤ 0.05) after 25 HBO procedures. Granulocyte count decreased by approx. 21% (*P* ≤ 0.05), and granulocyte percentage by 11.8% (*P* ≤ 0.01). In turn, the percentage of lymphocytes and monocytes increased after the treatment by 16.6% (*P* < 0.05) and 16.4% (*P* < 0.05), respectively.

**Conclusions:**

Exposure to HBO due to difficult-to-heal skin wounds does not significantly affect the levels of oxidative stress in the peripheral blood of patients and, from the point of view of oxidation–reduction processes, appears to be a safe therapeutic method for the treatment of chronic wounds.

## 1. Introduction

Chronic wounds are defined as wounds which require more than 8 weeks to heal despite optimal local treatment [1]. In industrialised countries, approx. 1–1.5% of the population is at risk of developing a chronic wound [2]. These wounds do not undergo the normal phases of wound healing in an orderly and timely manner [3]. Despite differences, chronic wounds have some common characteristics, e.g., increased levels of growth factors that can control cell migration, enzyme expression, and differentiation, proinflammatory cytokines that can regulate cell activity and functions [4], as well as reactive oxygen species (ROS) that have both defensive and signalling roles [3]. In local wound treatment, the TIME strategy developed by the European Wound Management Association is used, which contributes to the stimulation of natural healing mechanisms and includes: T—tissue debridement, I—infection and inflammation control, M—moisture balance, and E—epidermisation stimulation [5]. More advanced therapies, such as topical platelet-derived growth factor (PDGF), negative-pressure wound therapy, or bioengineered cell-containing therapies [6], are used to treat wounds that do not improve over a few weeks.

Chronic wound therapy may also include hyperbaric oxygen (HBO) use. Among the benefits of this form of therapy are increased oxygenation of hypoxic and ischaemic tissues, improved blood circulation, reduced oedema, and accelerated healing. HBO also has a bactericidal and bacteriostatic effect [7–9]. During the procedure, the patient is placed in a hyperbaric chamber and provided 100% oxygen for breathing [10], which increases the partial pressure of oxygen in the lungs, increases plasma oxygen concentration compared with normal conditions, and significantly increases the oxygen diffusion radius from capillaries to the surrounding tissues [11]. Hyperoxic condition has been shown to temporarily increase intracellular ROS and reactive nitrogen species (RNS) levels, which may promote certain processes associated with wound healing, such as proliferation and wound remodeling [12, 13]. Recently, hydrogels which rapidly generate molecular oxygen up to hyperoxic levels have been employed in wound treatment [13].

Generation of ROS, including free oxygen radicals, can lead to both beneficial and adverse effects, which depends on their concentration and intracellular location [14]. ROS are generated as natural by-products of metabolism, and their primary source in cells is the respiratory chain. In the process of cellular respiration, some oxygen naturally undergoes incomplete reduction, which leads to the formation of ROS [15]. Breathing 100% oxygen increases ROS generation in the body.

Thiobarbituric acid reactive substances (TBARS), the most prevalent substrate of which is malondialdehyde (MDA), secondary lipid peroxidation product, are one of the markers of oxidative stress. It has been demonstrated that an increase in their concentration may be indicative of oxidative damage to cell membranes [16]. Among the body's defences against excessive ROS generation are antioxidant enzymes, such as catalase (CAT), superoxide dismutase (SOD), and glutathione peroxidase (GPx) [17].

Cellular and molecular wound healing mechanisms are very complex, and their disturbances have not been fully understood. Moreover, it is not clearly known how hyperbaric oxygen affects the systemic oxidant–antioxidant equilibrium in the process of wound healing. Few studies looking at this problem have been conducted, and sometimes conflicting results have been obtained. Therefore, it seems reasonable to undertake research in this area. The aim of the study was to evaluate the TBARS concentration in the erythrocytes and blood plasma, as well as the activity of selected antioxidant enzymes: CAT, SOD, and GPX, in the erythrocytes of people with difficult-to-heal skin wounds undergoing HBO treatment. Moreover, selected peripheral blood counts were determined.

## 2. Materials and Methods

### 2.1. Patient Population and Study Design

The study included 23 patients (mean age 45.7 ± 16.3 years) of the Mazovian Centre for Hyperbaric Therapy and Wound Treatment in Warsaw Poland, undergoing HBO treatment due to the occurrence of difficult-to-heal skin wounds following mechanical injuries (in traffic accidents and other circumstances).

People with health problems characterised by proven oxidative stress were excluded from the study (cardiovascular disease, atherosclerosis, hypertension, but particularly diabetes mellitus). The patients were asked to abstain from drinking alcohol, smoking, and taking vitamins and other preparations that could affect the oxidant–antioxidant equilibrium during the experiment. The patients provided their written consent for the participation in the experiment. The study was approved by the Bioethics Committee of Ludwik Rydygier Collegium Medicum in Bydgoszcz, Nicolaus Copernicus University in Torun, Poland (approval no.: KB 260/2016).

**Figure 1:**
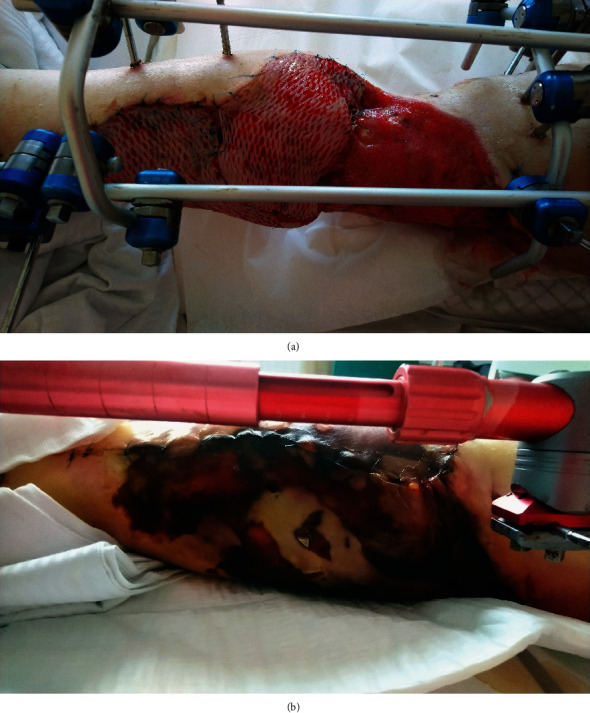
Lower leg wound was caused by a communication accident (a) before treatment in the hyperbaric chamber, (b) after 25 procedures in the hyperbaric chamber.

The patients underwent 25 HBO therapy procedures in the Haux Starmed 220 hyperbaric chamber which created reproducible environmental conditions (temperature, pressure, humidity, and ability to breathe pure oxygen for the same amount of time). The pressure inside the chamber during the procedure was 0.25 MPa, and the patients breathed 100% oxygen in three 20-minute cycles separated by two 5-minute intervals during which they breathed the air filling the chamber. Moreover, two 10-minute compression and decompression periods were included. During the experiment, local treatment in all patients was the same and was not changed. Ready-to-use hydrocolloid dressings were used on wounds. Following the HBO procedures, healing and reduction of wound size (Figures 1(a) and 1(b)) were observed in the patients.

### 2.2. Biochemical Analysis

Blood for biochemical analyses was collected from the basilic vein at three time points: before the first HBO procedure, approx. 5 min after the first procedure, and after a series of 25 procedures. The concentration of TBARS was determined in erythrocytes and blood plasma. The activities of the main three antioxidant enzymes, CAT, SOD, and GPx, were determined in erythrocytes. Moreover, prior to the first HBO procedure and after 25 procedures, selected peripheral blood counts were determined using th**e** Orphee - Mythic 22AL haematology analyser. All biochemical analyses were conducted in a laboratory at the Department of Medical Biology and Biochemistry of the Ludwik Rydygier Collegium Medicum in Bydgoszcz, Nicolaus Copernicus University in Torun, Poland.

#### 2.2.1. TBARS Concentration Measurement

The TBARS concentration was measured using the method by Buege and Aust [18] as modified by Esterbauer and Cheeseman [19]. Lipid peroxidation products were identified using thiobarbituric acid (TBA). The main lipid peroxidation product that reacts with thiobarbituric acid is MDA; therefore, for the sake of simplicity, the levels of TBARS were expressed as the concentration of MDA. The MDA concentration in erythrocytes was expressed in nmol MDA/g Hb, and that in blood plasma was expressed in nmol MDA/mL of plasma.

#### 2.2.2. CAT Activity Measurement

The CAT activity was determined by measuring the decrease in the absorbance of a solution of hydrogen peroxide (H_2_O_2_) decomposed by this enzyme. The decrease in the absorbance value is directly proportional to the reduction of the H_2_O_2_ concentration in the solution [20]. The CAT activity was expressed in IU/g Hb.

#### 2.2.3. SOD Activity Measurement

Determination of the SOD activity was based on the inhibition of adrenaline autoxidation to adrenochrome in alkaline conditions. To measure the SOD activity, a previously obtained haemolysate after removal of haemoglobin with a chloroform–ethanol mixture was used. Centrifugation generated two layers: the upper layer containing the enzyme and lower layer containing denatured haemoglobin and chloroform [21]. The SOD activity was determined by continuous recording of the reaction using a reaction kinetics programme on a Varian spectrophotometer and expressed in U/g Hb.

#### 2.2.4. GPx Activity Measurement

The GPx activity was determined at 20°C using a method based on the decomposition of hydrogen peroxide by the enzyme with the concurrent oxidation of reduced glutathione [22]. The results were expressed in U/g Hb.

### 2.3. Statistical Analysis

The study results were presented as means with standard deviation (SD) values. To assess the normal distribution of the data, the Kolmogorov–Smirnov test was used. Statistical analysis of the oxidative stress parameters was performed using analysis of variance (ANOVA, Bonferroni post hoc test) (*STATISTICA v.* 9.1). Student's *t*-test for variables in paired measurements was used to compare mean values calculated for the blood counts. Dependencies between the analysed parameters were assessed using correlation matrices. A statistical hypothesis of the significance of correlation coefficients (*r*) was tested. Differences at significance level *P* ≤ 0.05 were presumed as statistically significant. Results close to but higher than *P* = 0.05 may indicate certain tendencies and can be an inspiration for further research. In this study, such results are presented as being at the level of statistical tendency. The threshold of statistical tendency was established at *P* = 0.09.

## 3. Results

### 3.1. TBARS Level

No statistically significant changes in the TBARS concentration in erythrocytes and blood plasma were observed (Table 1). However, there was a certain tendency to change. The plasma TBARS concentration after 25 procedures decreased by 10.5% compared to that measured before the first procedure (*P* > 0.05).

### 3.2. Antioxidant Enzyme Activities

No statistically significant changes in the CAT, SOD, and GPx activities in the erythrocytes of the study participants were determined (Table 1). However, a tendency to change was observed in the erythrocyte CAT activity. The activity of this enzyme in erythrocytes after the first HBO procedure decreased by 3% compared with that before the procedure (*P* > 0.05).

**Figure 2:**
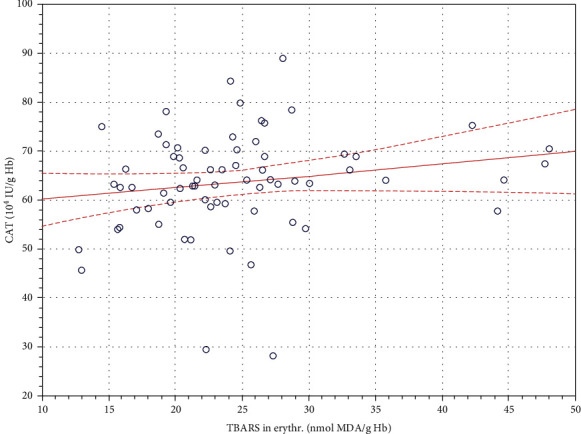
Linear regression of the catalase (CAT) activity versus the thiobarbituric acid reactive substances (TBARS) concentration in the erythrocytes of patients with difficult-to-heal skin wounds before initiation of treatment in a hyperbaric chamber (*r* = 0.394; *P* ≤ 0.05).

A statistically significant positive correlation between the CAT activity and the TBARS concentration in the erythrocytes of patients was found prior to treatment in the hyperbaric chamber (*r* = 0.394; *P* ≤ 0.05) (Figure 2).

### 3.3. Blood Counts

A statistically significant decrease in platelet count by 18.7% (*P* ≤ 0.05) was observed in peripheral blood after 25 procedures in hyperbaric chamber (Table 2). Moreover, granulocyte count decreased by approx. 21% (*P* ≤ 0.05), and granulocyte percentage by 11.8% (*P* ≤ 0.01). In turn, the percentage of lymphocytes and monocytes increased by 16.6% (*P* < 0.05) and 16.4% (*P* < 0.05), respectively, after treatment completion. No statistically significant changes were observed for the remaining peripheral blood counts.

## 4. Discussion

Oxygen plays a key role in wound healing. This role has been demonstrated in processes such as oxidative killing of microorganisms, collagen synthesis, angiogenesis, and neovascularisation [23]. It has been proven that a higher oxygen supply can increase the capacity of epithelial cells for mitotic divisions [24]. Chronic wounds are often characterised by excessive ROS generation due to the absence of antioxidants, such as vitamins E, C, and A. The level of antioxidants has been proven to decrease with age, resulting in a delayed healing response in older people [23]. The precise role of oxygen in wound healing under hyperbaric oxygen conditions has not yet been clearly defined.

In the presented study, no statistically significant changes in the TBARS concentration were observed in both erythrocytes and blood plasma, which suggests that the use of HBO does not significantly affect the intensity of lipid peroxidation measured in the peripheral blood of patients with chronic wounds. Hyperbaric oxygen therapy increases oxygenation of tissues [25], but despite the increased generation of ROS directly caused by the increased amount of oxygen reaching the cells, there was no change in the oxidant–antioxidant equilibrium. Only a tendency of TBARS to decrease in blood plasma after 25 HBO procedures was seen.

A lack of increase of oxidative stress after HBO procedures was also demonstrated by Corcoran et al. [26] in patients with osteonecrosis by measuring plasma concentrations of F_2_-isoprostanes and isofurans. A statistically significant decrease in plasma MDA was reported by Sureda et al. [27] in patients with chronic wounds after 20 HBO procedures. The authors also reported a significantly lower level of this secondary lipid peroxidation product one month after the wound had healed compared with the initial concentration. In turn, in the ulcer tissue of patients with diabetic foot ulcer, an increased MDA concentration was demonstrated on the 14th day of HBO treatment compared with that in the control group—patients on standard treatment not including HBO [28]. In other studies, increased MDA was observed in the blood plasma of patients with diabetic foot only after the first HBO procedure compared with the concentration measured before it, whereas after completion of 15 HBO procedures, the level of this lipid peroxidation product was unchanged vs. baseline. In turn, the levels of 8-isoprostane and advanced oxidation protein products (AOPPs) increased in a statistically significant manner vs. baseline only after completion of 15 HBO procedures [29]. In our earlier study in patients with different conditions after hyperbaric oxygen therapy, there were no statistically significant changes in the TBARS concentration in both blood plasma and erythrocytes [30].

However, there are publications which demonstrate that long-term exposure to high oxygen concentrations, in the form of HBO, induces generation of ROS which results in cell damage. Alleva et al. [31] demonstrated a beneficial effect of *α*-lipoic acid in patients with chronic wounds treated with HBO. The authors showed that supplementation of this antioxidant reduces oxidation of lipids and DNA caused by oxygen exposure, which can promote the beneficial effects of HBO treatment. Increased lipid peroxidation after HBO procedures has been suggested by some studies conducted in animals. For example, Giulivi et al. [32] showed an increase in the MDA concentration in the lungs after HBO procedures in rats, while in another study, Liu et al. [33] recorded an increased TBARS level in the cell membrane of erythrocytes of female rats with experimentally induced diabetes. It appears that further studies are necessary for a clear identification of the effect of HBO on the lipid peroxidation process.

Prior to the HBO treatment in the hyperbaric chamber, we found in patients a statistically significant positive correlation between the CAT activity and the TBARS concentration in erythrocytes (*r* = 0.394; *P* ≤ 0.05), which could indicate a disturbance in the oxidant–antioxidant mechanisms in the course of healing of difficult wounds. There were no statistically significant changes in the activity of the investigated antioxidant enzymes in erythrocytes, which confirms a lack of a significant effect of hyperbaric oxygen on the course of oxidation–reduction processes. The study only showed a certain tendency to decrease the catalase activity after the first HBO procedure (Table 1). It seems that the repeatability of HBO procedures can increase the tolerance of mechanisms which determine the oxidant–antioxidant equilibrium to increased ROS generation. Results similar to those presented in this study were obtained by Sureda et al. [27] who reported no statistically significant changes in the CAT, SOD, GPx, as well as glutathione reductase activities in the erythrocytes of patients with chronic wounds.

In turn, a statistically significant reduction in the CAT activity in erythrocytes after an HBO procedure was shown in our previous study in which patients with different conditions had received HBO treatment no more than three times in their lives at the time of the experiment. However, no statistically significant changes in the CAT activity were observed in the erythrocytes of people treated with HBO multiple times prior to the experiment, as well as no significant changes in the SOD activity were found in the erythrocytes of all study participants, regardless of the number of prior procedures in the hyperbaric chamber. Also, there were no changes in the GPx activity in erythrocytes following a procedure in the hyperbaric chamber, but the activity of this enzyme prior to treatment in patients who had previously used hyperbaric chamber many times was significantly lower than in patients who had received HBO treatment no more than three times [30]. In patients with sudden sensorineural hearing loss, a decrease in the CAT activity in erythrocytes was observed after the first procedure in the hyperbaric chamber, the SOD activity in erythrocytes decreased after the 14th procedure, and the GPx activity increased after the 14th procedure [34].

In a study by other authors, a statistically significant increase in the CAT activity in the blood plasma of patients with chronic wounds was demonstrated after both the first and the fifth day of HBO treatment compared with the activities determined before each of these procedures [27]. After the 20th procedure, there was an increase in the CAT activity as well, but it was not statistically significant. However, the authors did not identify any statistically significant changes in the SOD activity in blood plasma. Ma et al. [28], on the 14th day of their experiment, showed higher CAT and SOD activities in ulceration specimens derived from patients treated with HBO (twice daily for 90 minutes at 2.5 atm for 2 weeks) due to skin wound ulcers of the foot caused by diabetes, compared with such activities in people treated conventionally without the use of the hyperbaric chamber. At the same time, a significant improvement was observed in the healing of foot wounds following HBO treatment. In turn, studies in rats proved that repeated treatment with HBO for 1 hour increases the expression of the CAT gene and activity of the enzyme in the cardiac muscle after ischaemia [35].

Blood counts revealed a statistically significant decrease in the number of platelets in patients after 25 procedures in hyperbaric chamber (Table 2). Other statistically significant changes in blood counts were related to the number and percentage of granulocytes which decreased after completion of the HBO procedures. Conversely, the percentages of lymphocytes and monocytes increased significantly. No statistically significant changes were observed for the remaining peripheral blood counts. Concurrently, all investigated blood count values were within their respective normal ranges. A statistically insignificant reduction of the platelet count in patients after HBO procedures was reported by Gunes and Aktas [36]. The findings described by these authors, similarly to ours presented here, included no statistically significant effect of HBO treatment on haemoglobin, haematocrit, red blood cells, mean corpuscular volume, mean corpuscular haemoglobin, red blood cell distribution width, mean corpuscular haemoglobin concentration, platelet distribution width, and mean platelet volume. Studies by other authors showed a statistically insignificant reduction of haematocrit and erythrocyte counts after 20 HBO procedures in patients with various pathologies associated with hypoxia [37].

The statistically significant decrease in platelet count observed in the present study may be associated with a certain degree of normalisation of coagulation processes under increased oxygenation of tissues during exposure to HBO. In a study of healthy lowland volunteers who remained under hypoxic conditions at the height of 5200 m above sea level, researchers showed an increase in platelet count and fibrinogen concentration on the 7th day of the experiment as a result of acclimatisation [38]. However, during a prolonged stay at a high altitude, the platelet count in healthy individuals was observed to decrease after 3 and 13 months compared with the values observed at sea level [39]. Changes in the number and/or percentage of leukocytes demonstrated in this study may result from an anti-inflammatory effect of hyperbaric oxygen treatment. Grimberg-Peters et al. [40], among other groups, showed a supportive effect of HBO in an analysis of the effect of such a treatment aimed at reducing inflammation on the activity of neutrophils isolated from severely injured patients (days 1–2 after trauma). In turn, a statistically significant decrease in the number of leukocytes after therapy combined with HBO compared with baseline was observed by Irawan et al. [41] in patients with a diabetic foot ulcer.

This study has some limitations. It could include more parameters, which characterize the redox equilibrium, and concern the injured tissue/tissues. However, the first issue is always depended on study funding, while the second one, in the case of such wounds, in such patients, raises ethical questions. However, first of all, this study included a small group of patients; therefore, the obtained results should be considered as preliminary to further studies in that area.

## 5. Conclusions

Analysis of the obtained results shows that the effect of hyperbaric oxygen on the oxidation–reduction processes is not clear. However, the lack of significant changes suggests that HBO therapy does not increase systemic oxidative stress and, from the point of view of maintaining the oxidant–antioxidant equilibrium, it seems to be a safe therapeutic method for the treatment of chronic wounds.

## Figures and Tables

**Table 1:** The concentration of thiobarbituric acid reactive substances (TBARS) in erythrocytes and blood plasma, and the activities of catalase (CAT), superoxide dismutase (SOD), and glutathione peroxidase (GPx) were determined in patients with difficult-to-heal skin wounds receiving hyperbaric oxygen (HBO) treatment.

Determined parameter HBO	Before procedure	After 1st HBO procedure	After 25th HBO procedure
TBARS er. (nmol MDA/g Hb)	25.36 ± 7.94	23.87 ± 7.57	25.11 ± 7.89
TBARS pl. (nmol MDA/mL)	0.57 ± 0.15	0.55 ± 0.15	0.51 ± 0.14
CAT (10^4^IU/g Hb)	64.06 ± 10.47	62.14 ± 11.90	65.51 ± 6.59
SOD (U/g Hb)	626.49 ± 85.04	732.31 ± 88.74	732.31 ± 82.57
GPx (U/g Hb)	8.58 ± 4.10	7.40 ± 4.63	7.08 ± 4.00

TBARS er.: thiobarbituric acid reactive substances in erythrocytes; TBARS pl.: thiobarbituric acid reactive substances in plasma; CAT: catalase; SOD: superoxide dismutase, GPx: glutathione peroxidase. Results are expressed as Mean ± SD. No statistically significant differences.

**Table 2:** Selected peripheral blood counts of patients with difficult-to-heal wounds treated with hyperbaric oxygen (HBO).

Determined parameter	Before procedure	After 25th HBO	Normal range procedure
HGB (g/dl)	13.94 ± 1.99	13.65 ± 1.99	11.0–17.0
HCT (%)	38.24 ± 5.06	40.48 ± 12.21	35.0–55.0
RBC (10^6^/*μ*l)	4.24 ± 0.46	4.09 ± 0.53	4.0–6.20
PLT (10^3^/*μ*l)	368.26 ± 136.51	299.33 ± 130.57^∗^	150–400
WBC (10^3^/*μ*l)	8.21 ± 3.09	7.05 ± 1.89	4.0–12.0
LYM (%)	27.88 ± 8.37	32.5 ± 7.76^∗^	25.0–50.0
MON (%)	7.06 ± 1.7	8.22 ± 1.8^∗^	2.0–10.0
GRA (%)	62.29 ± 9.43	54.91 ± 6.99^∗∗^	50.0–80.0
EOS (%)	2.88 ± 1.72	3.38 ± 2.25	0.0–5.0
BAS (%)	0.44 ± 0.21	0.45 ± 0.17	0.0–2.0
LYM (10^3^/*μ*l)	2.1 ± 0.55	2.28 ± 0.37	1.0–5.0
MON (10^3^/*μ*l)	0.56 ± 0.21	0.6 ± 0.18	0.1–1.0
GRA (10^3^/*μ*l)	5.16 ± 2.61	4.08 ± 1.27^∗^	2.0–8.0
EOS (10^3^/*μ*l)	0.22 ± 0.16	0.26 ± 0.19	0.0–0.40
BAS (10^3^/*μ*l)	0.0095 ± 0.03	0.0048 ± 0.2	0.0–0.2
MCHC (g/dl)	35.12 ± 2.96	33.77 ± 6.9	31.0–35.50
MCH (pg)	32.99 ± 4.52	33.46 ± 2.84	26.0–34.0
MCV (fl)	92.64 ± 7.96	90.75 ± 15.08	80.0–100.0
RDW (%)	15.33 ± 1.36	15.31 ± 1.21	10.0–16.0
MPV (fl)	8.38 ± 0.92	8.7 ± 1.03	7.0–10.5
PCT (%)	0.31 ± 0.1	0.27 ± 0.1	0.20–0.50
PDW (%)	13.59 ± 1.09	13.91 ± 1.23	10.0–18.0

HGB: haemoglobin; HCT: haematocrit; RBC: erythrocytes; PLT: platelets; WBC: leukocytes; LYM: lymphocytes; MON: monocytes; GRA: granulocytes; EOS: eosinophils; BAS: basophils; MCHC: mean haemoglobin concentration in red blood cells; MCH: mean haemoglobin mass in red blood cells; MCV: mean red blood cell volume; RDW: red blood cell distribution width; MPV: mean platelet volume; PCT: plateletcrit; PDW: platelet distribution width. Values are expressed as means ± standard deviations of the means. ^∗^Statistically significant difference compared with parameters measured before the HBO therapy (^∗^*P* ≤ 0.05; ^∗∗^*P* ≤ 0.01).

## Data Availability

The study data used to support the findings of this study are included within the article.

## References

[1] Englisz-Jurgielewicz B., Cholewka A., Firganek E. (2020). Evaluation of hyperbaric oxygen therapy effects in hard-to-heal wounds using thermal imaging and planimetry. *Journal of Thermal Analysis and Calorimetry*.

[2] Gottrup F., Appelqvist J., Price P. (2010). Outcomes in controlled and comparative studies on non-healing wounds: recommendations to improve the quality of evidence in wound management. *Journal of Wound Care*.

[3] Frykberg R. G., Banks J. (2015). Challenges in the treatment of chronic Wounds. *Advances in Wound Care*.

[4] Diegelman R. F., Evans M. C. (2004). Wound healing: an overview of acute, fibrotic and delayed healing. *Frontiers in Bioscience*.

[5] Mościcka P., Szewczyk M. T., Cwajda-Białasik J., Jawień A. (2019). The role of compression therapy in the treatment of venous leg ulcers. *Advances in Clinical and Experimental Medicine*.

[6] Krzyszczak P., Schloss R., Palmer A., Berthiaume F. (2018). The role of macrophages in acute and chronic wound healing and interventions to promote pro-wound healing phenotypes. *Frontiers in Physiology*.

[7] Gębala-Prajsnar K., Stanek A., Pasek J. (2015). Selected physical medicine interventions in the treatment of diabetic foot syndrome. *Acta Angiologica*.

[8] Glik J., Cholewka A., Stanek A. (2019). Thermal imaging and planimetry evaluation of the results of chronic wounds treatment with hyperbaric oxygen therapy. *Advances in Clinical and Experimental Medicine*.

[9] Cholewka A., Knefel G., Stanek A. (2012). Thermal imaging and TC oximetry measurements of hyperbaric oxygen therapy (HBO) effects on trophic ulceration of the crura. *Journal of Thermal Analysis and Calorimetry*.

[10] Kranke P., Bennett M. H., Martyn-St James M., Schnabel A., Debus S. E., Weibel S. (2015). Hyperbaric oxygen therapy for chronic wounds. *The Cochrane Database Systematic Reviews*.

[11] Kawecki M., Knefel G., Szymańska B., Nowak M., Sieroń A. (2006). Aktualne wskazania i możliwości zastosowania hiperbarycznej terapii tlenowej [Present indications and capabilities of HBO applying]. *Borgis-Balneologia Polska*.

[12] Fosen K. M., Thom S. R. (2014). Hyperbaric oxygen, vasculogenic stem cells, and wound healing. *Antioxidants and Redox Signaling*.

[13] Park S., Park K. M. (2018). Hyperbaric oxygen-generating hydrogels. *Biomaterials*.

[14] Thom S. R. (2009). Oxidative stress is fundamental to hyperbaric oxygen therapy. *Journal of Applied Physiology*.

[15] Cedikova M., Pitule P., Kripnerova M., Markova M., Kuncova J. (2016). Multiple roles of mitochondria in aging processes. *Physiological Research*.

[16] Dalle-Donne I., Rossi R., Colombo R., Giustarini D., Milzani A. (2006). Biomarkers of oxidative damage in human disease. *Clinical Chemistry*.

[17] Gałecka E., Jackiewicz R., Mrowicka M., Florkowski A., Gałecki P. (2008). Antioxidant enzymes-structure, properties, functions. *Polski Merkuriusz Lekarski*.

[18] Buege J., Aust S. (1978). [30] Microsomal lipid peroxidation. *Methods in Enzymology*.

[19] Esterbauer H., Cheeseman K. (1990). [42] Determination of aldehydic lipid peroxidation products: Malonaldehyde and 4-hydroxynonenal. *Methods in Enzymology*.

[20] Beers R., Sizer J. (1952). Spectrophotometric method for measuring the breakdown of hydrogen peroxide by catalase. *Journal of Biological Chemistry*.

[21] Misra H. P., Fridovich I. (1972). The role of superoxide anion in the autoxidation of epinephrine and a simple assay for superoxide dismutase. *Journal of Biological Chemistry*.

[22] Paglia D. E., Valentine W. N. (1967). Studies on the quantitative and qualitative characterization of erythrocyte glutathione peroxidase. *The Journal of Laboratory and Clinical Medicine*.

[23] Schäfer M., Warner S. (2008). Oxidative stress in normal and impaired wound repair. *Pharmacological Research*.

[24] Constant J., Suh D., Hussain M., Hunt T. (1996). Wound healing angiogenesis: the metabolic basis of repair. *Molecular Cellular and Clinical Aspects of Angiogenesis*.

[25] Efrati S., Gall N., Bergan J. (2009). Hyperbaric oxygen, oxidative stress, NO bioavailability and ulcer oxygenation in diabetic patients. *Undersea and Hyperbaric Medicine*.

[26] Corcoran T., Ting S., Mas E. (2017). Hyperbaric oxygen therapy is not associated with oxidative stress assessed using plasma F2-isoprostanes and isofurans. *Prostaglandins, Leukotrienes, and Essential Fatty Acids*.

[27] Sureda A., Batle J. M., Martorell M. (2016). Antioxidant response of chronic wounds to hyperbaric oxygen therapy. *PLoS One*.

[28] Ma L., Li P., Shi Z., Hou T., Chen X., Du J. (2013). A prospective, randomized, controlled study of hyperbaric oxygen therapy: effects on healing and oxidative stress of ulcer tissue in patients with a diabetic foot ulcer. *Ostomy/Wound Management*.

[29] Gürdöl F., Cimşit M., Oner-Iyidoğan Y., Körpinar S., Yalçinkaya S., Koçak H. (2008). Early and late effects of hyperbaric oxygen treatment on oxidative stress parameters in diabetic patients. *Physiological Research*.

[30] Paprocki J., Sutkowy P., Krzyżyńska-Malinowska E., Piechocki J., Woźniak A. (2013). The indicators of oxidant-antioxidant balance in patients performed hyperbaric oxygenation. *Polish Hyperbaric Research*.

[31] Alleva R., Nasole E., Di Donato F., Borghi B., Neuzil J., Tomasetti M. (2005). *α*-Lipoic acid supplementation inhibits oxidative damage, accelerating chronic wound healing in patients undergoing hyperbaric oxygen therapy. *Biochemical and Biophysical Research Communications*.

[32] Giulivi C., Lavagno C., Lucesoli F., Bermudez M., Boveris A. (1995). Lung damage in paraquat poisoning and hyperbaric oxygen exposure: Superoxide- mediated inhibition of phospholipase a_2_. *Free Radical Biology and Medicine*.

[33] Liu D. Z., Chien S. C., Tseng L. P., Yang C. B. (2003). The influence of hyperbaric oxygen on hemorheological parameters in diabetic rats. *Biorheology*.

[34] Paprocki J., Sutkowy P., Piechocki J., Woźniak A. (2019). Markers of oxidant-antioxidant equilibrium in patients with sudden sensorineural hearing loss treated with hyperbaric oxygen therapy. *Oxidative Medicine and Cellular Longevity*.

[35] Kim C., Choi H., Chun Y., Kim G., Park J., Kim M. (2001). Hyperbaric oxygenation pretreatment induces catalase and reduces infarct size in ischemic rat myocardium. *Pflügers Archiv*.

[36] Gunes A. E., Aktas S. (2017). Effect of hyperbaric oxygen therapy on complete blood count. *Undersea and Hyperbaric Medicine*.

[37] Sinan M., Ertan N. Z., Mirasoglu B. (2016). Acute and long-term effects of hyperbaric oxygen therapy on hemorheological parameters in patients with various disorders. *Clinical Hemorheologya nd Microcirculation*.

[38] Rocke A. S., Paterson G. G., Barber M. T. (2018). Thromboelastometry and platelet function during acclimatization to high altitude. *Thrombosis and Haemostasis*.

[39] Vij A. G. (2009). Effect of prolonged stay at high altitude on platelet aggregation and fibrinogen levels. *Platelets*.

[40] Grimberg-Peters D., Büren C., Windolf J., Wahlers T., Paunel-Görgülü A. (2016). Hyperbaric oxygen reduces production of reactive oxygen species in neutrophils from polytraumatized patients yielding in the inhibition of p 38 MAP kinase and downstream pathways. *PLoS One*.

[41] Irawan H., Semadi I. N., Widiana I. G. R. (2018). A pilot study of short-duration hyperbaric oxygen therapy to improve HbA1c, leukocyte, and serum creatinine in patients with diabetic foot ulcer Wagner 3-4. *The Scientific World Journal*.

